# A multigene family encoding surface glycoproteins in *Trypanosoma congolense*

**DOI:** 10.15698/mic2017.03.562

**Published:** 2017-03-02

**Authors:** Magali Thonnus, Amandine Guérin, Loïc Rivière

**Affiliations:** 1 Fundamental Microbiology and Pathogenicity Unit, CNRS UMR 5234, Bordeaux University, France.; 2 Current affiliation: CNRS UMR 5235, Montpellier 2 University, France.

**Keywords:** Trypanosoma congolense, surface glycoprotein, multigene family, lectin-like, trypanosomes

## Abstract

*Trypanosoma congolense*, the causative agent of the most important livestock disease in Africa, expresses specific surface proteins involved in its parasitic lifestyle. Unfortunately, the complete repertoire of such molecules is far from being deciphered. As these membrane components are exposed to the host environment, they could be used as therapeutic or diagnostic targets. By mining the *T. congolense* genome database, we identified a novel family of lectin-like glycoproteins (TcoClecs). These molecules are predicted to have a transmembrane domain, a tandem repeat amino acid motif, a signal peptide and a C-type lectin-like domain (CTLD). This paper depicts several experimental arguments in favor of a surface localization in bloodstream forms of *T. congolense*. A TcoClec gene was heterologously expressed in U-2 OS cells and the product could be partially found at the plasma membrane. TcoClecs were also localized at the surface of *T. congolense* bloodstream forms. The signal was suppressed when the cells were treated with a detergent to remove the plasma membrane or with trypsin to « shave » the parasites and remove their external proteins. This suggests that TcoClecs could be potential diagnostic or therapeutic antigens of African animal trypanosomiasis. The potential role of these proteins in *T. congolense* as well as in other trypanosomatids is discussed.

## INTRODUCTION

Trypanosomes are eukaryotic microbes found in different parts of the world. In Africa, these deadly parasites are responsible for neglected diseases called sleeping sickness in human (*Trypanosoma brucei gambiense* and *Trypanosoma brucei rhodesiense*) 1 and nagana in cattle and game animals (*Trypanosoma brucei brucei*, *Trypanosoma congolense* and *Trypanosoma vivax*) 2,3.

The development of trypanosomes follows a complex lifecycle. Bloodstream forms (BSF) proliferate in the blood of the infected mammalian host and are ingested by an insect (called tsetse fly, *Glossina* spp) during the meal. Then, they differentiate into procyclic forms (PCF) in the midgut and migrate to the salivary glands and proboscis where they attach as epimastigote forms (EMF). Finally, they differentiate into infective metacyclic forms (MCF) that are transmitted to a new mammalian host during the next blood meal.

Trypanosomes have become an interesting model to study biological processes. For example, they possess glycosomes, which are specialized peroxysomes involved in glycolysis, a unique tubular mitochondrion, and a flagellar pocket that is the only site for endo- and exocytosis 4,5. Moreover, RNA editing, glycophosphatidylinositol (GPI) anchoring, trans-splicing and antigenic variation are biological phenomena that were initially discovered in these parasites 6,7,8.

*T. b. brucei* is widely used as a model organism in African trypanosome biology. On the contrary, *T. congolense*, the main causative agent of African animal trypanosomiasis, is poorly studied.

These two species have different behavior upon *in vivo* infection and *in vitro* cultivation. In the mammalian host, *T. congolense* adheres to endothelial cells and red blood cells, whereas *T. b. brucei* does not 9. Interestingly, *in vitro* *T. congolense* BSF adhere directly to the flask but not *T. b. brucei*. This could reflect a different composition or property of the plasma membrane. Actually, in both species, the entire surface is covered by millions of copies of a single variant surface glycoprotein (VSG) which constitute a « coat » that masks other antigens and fools the immune system of the host 10,11. Nevertheless, VSG are not responsible for adhesion and the repertoire of surface proteins is not completely well-known. Our study aimed to discover such new molecules in *T. congolense *BSF.

As lectins are exposed on the surface of cells, we performed a search in the African trypanosomes genome database in order to identify candidates. We found a family of proteins that possesses several features of surface molecules, i.e. a transmembrane domain, a signal peptide, a tandem repeat amino acid motif and a C-type lectin fold domain 12,13. We called these proteins TcoClecs. Interestingly, these molecules have already been detected in the cell-surface phylome 14, and a recent study has shown that TcoClec orthologs in *T. b. brucei* are glycoproteins retained in the endoplasmic reticulum (ER) 15.

Here we report that TcoClecs are exposed on the surface of *T. congolense* BSF.

## RESULTS

### *In silico* identification of new putative *T. congolense* lectins

**Figure 1:**
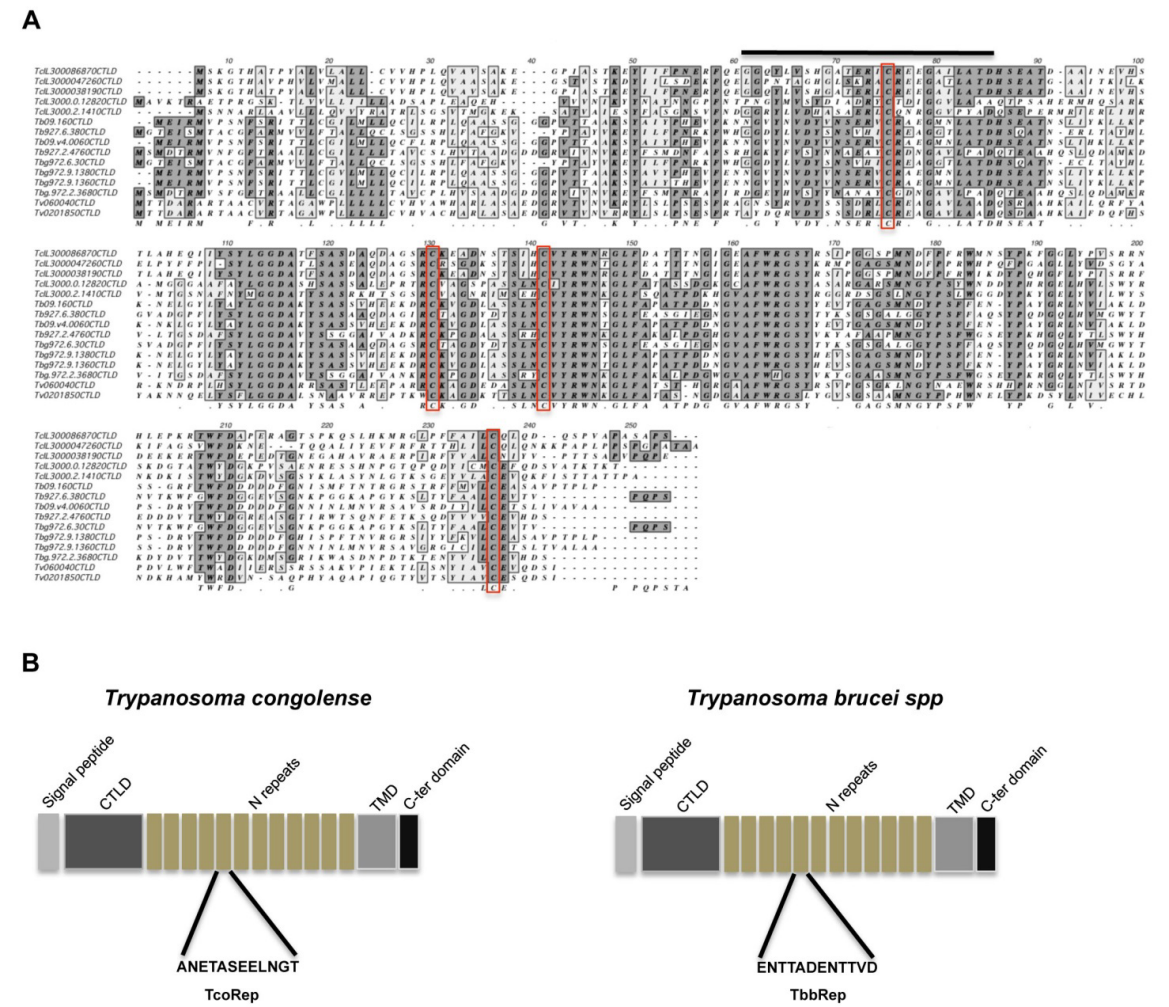
FIGURE 1: Comparison of Clec proteins in African trypanosomes. **(A)** Alignment of C-type lectin-like domain (CTLD) from African trypanosomes Clec proteins. Sequences were extracted and aligned using MacVector V11. Putative critical cysteines important for protein folding are indicated (red boxes) as well as link module (black line). Dark grey boxes contain identical residues, light grey boxes contain conservative changes. TcIL3000, *Trypanosoma congolense* IL3000 (reference strain); Tb, *Trypanosoma brucei brucei*; Tbg, *Trypanosoma brucei gambiense*; Tv, *Trypanosoma vivax*. **(B)** General features shared by Clec proteins in *T. congolense* and *T. b. brucei*. TMD, transmembrane domain.

Our first goal was to identify *in silico* new genes that could code for surface proteins of *T. congolense *BSF. In order to minimize the number of potential candidates, we focused our search on lectins. We chose the C-type lectin-like domain (CTLD, InterPro IPR016187) because in metazoan parasites, lectins that contain this motif are involved in host-parasite interaction 16. In protozoans, this domain was only described in the human pathogen *Cryptosporidium parvum* 17. We restricted our search to multigene families of proteins with a transmembrane domain and a signal peptide. These last features are common to many unicellular surface proteins 18. By mining the *T. congolense* genome with the Tritryp website (Tritrypdb.org), we found genes (see the materials and methods section) corresponding to a unique family. Interestingly, this family was already identified in the cell-surface phylome as « Fam77 » « Lectin-like membrane protein » 14. Also, orthologs in *T. b. brucei* have been described recently and are called TbIGP (invariant glycoproteins) 15. This family could be divided in subfamilies according to phylogenetic analysis 15. Alignment of African trypanosomes CTLDs revealed both conserved and variable regions. Four cysteine residues are conserved and could be essential for correct folding. In addition, a link module important for carbohydrate recognition is present (Figure 1A) 17,19.

Strikingly, these proteins have a tandem repeat amino acid motif. The sequence of this motif is different between *T. congolense* and *T. b. brucei* (Figure 1B). We decided to name these proteins TcoClecs according to current nomenclature 13,17.

### TcoClecs can be heterologously expressed on the surface of U-2 OS cells

**Figure 2:**
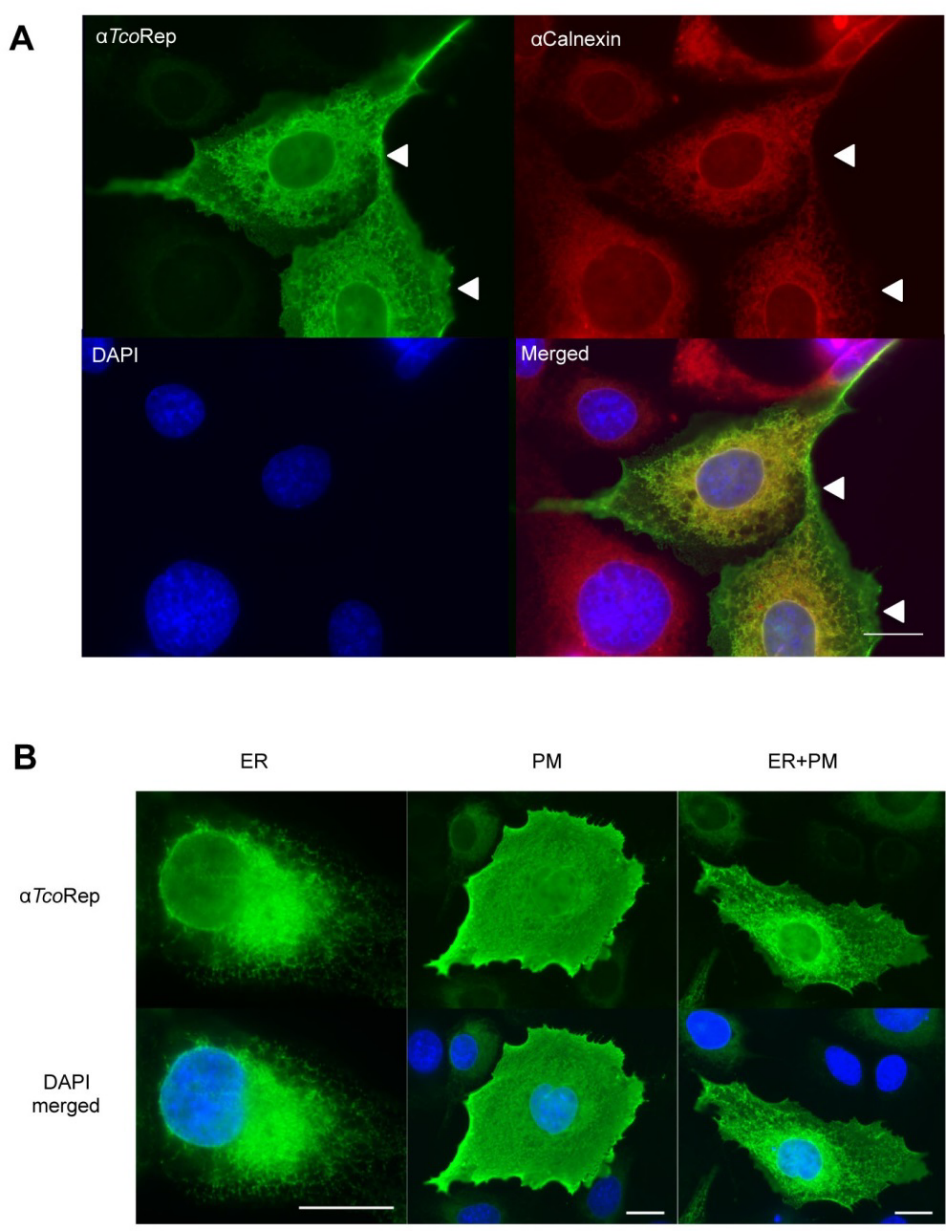
FIGURE 2: Immunofluorescence analysis of U-2 OS cells expressing a TcoClec protein. U-2 OS cells expressing a TcoClec protein are indicated (white arrowheads). **(A)** Cells were stained with anti-TcoRep, anti-calnexin and DAPI. Merged picture (bottom right) shows that TcoClec and calnexin colocalize partially. **(B)** Three patterns could be observed in the same proportions: endoplasmic reticulum (ER, left), plasma membrane (PM, middle) and both localizations (right). Cells were stained with anti-TcoRep and DAPI. Bar, 20 µm.

We employed polyclonal antibodies directed against the amino acid motif (anti TcoRep, Figure 1B) to characterize further these molecules. As protein expression in heterologous cells can help to decipher localizations 20, we used this strategy to first prove the specificity of our antibodies. U-2 OS cells do not possess any TcoClec orthologs and are well-suited for heterologous expression of trypanosomal proteins 21,22,23. From Figure 2, it can be seen that our antibodies react only with transfected cells, whereas the control marker (calnexin) is detected in all cells. Interestingly, TcoClec partially colocalizes with calnexin, suggesting that the protein could be distributed in the ER. In addition, some signal is seen on the edge of the transfected cells. This could correspond to a plasma membrane localization (Figure 2A). Moreover, three localization patterns are observed: ER, plasma membrane and both ER and plasma membrane (Figure 2B). These results suggest that in U-2 OS cells, heterologously-expressed TcoClec can be directed to the membrane. Finally, these experiments validate our antibodies as a specific tool for immunofluorescence assay (IFA).

### TcoClecs are membrane glycoproteins exposed at the surface of *T. congolense* BSF

**Figure 3:**
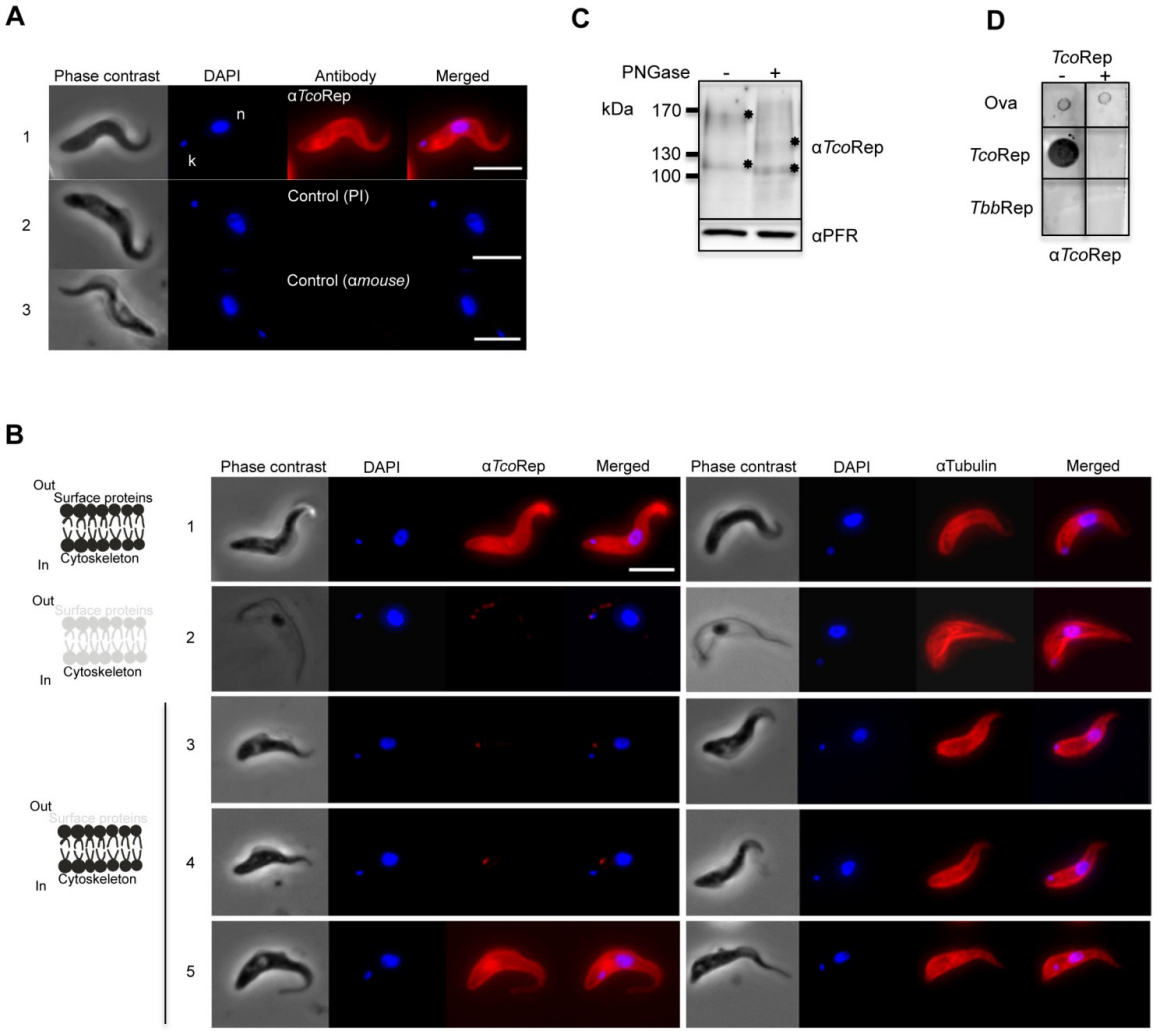
FIGURE 3: Expression and cellular localization of TcoClec in BSF of *T. congolense*. **(A)** Immunofluorescence analysis of *T. congolense* BSF. Cells were stained with mouse anti-TcoRep (1); control pre-immune serum (2); or secondary antibody alone (3). DAPI was used to stain both nucleus (n) and kinetoplast (k). Phase contrast images are shown on the left. **(B)** Immunofluorescence analysis. Images are typical examples showing phase contrast, DAPI staining of the nucleus and the kinetoplast, and staining with anti-TcoRep or anti-tubulin. Whole-cell *T. congolense* BSF (1); cytoskeleton-extracted cells (treated with NP40) (2); and trypsin-treated cells (« shaved » parasites) with IFA performed immediately (3), 5 h (4) and 14 h (5) after the treatment. A schematic illustration is shown on the left to explain the effects of the treatments on Tco BSF. Bar, 5 µm. **(C)** Western blot analysis. Tco BSF cell lysates treated (+) or not (-) with PNGase F. Mouse anti-TcoRep and rabbit anti-PFR (control) were used. Asterisks show band shifts. **(D)** Peptide competition assay (PCA). Dot blot analysis developed with anti-TcoRep antibodies. Anti-TcoRep antibodies were incubated (+) or not (-) with the TcoRep peptide. Ovalbumin (Ova), TcoRep peptide and TbbRep peptide were spotted on the filters.

Figure 3A shows that anti-TcoRep stains the whole cell, with a marked signal at the periphery. It is a typical plasma membrane staining. No signal could be obtained with control antibodies (pre-immune and secondary antibody alone). This result suggests that TcoClecs could be distributed on the plasma membrane. Unfortunately, there is no *T. congolense* BSF surface marker available to see potential colocalization. To overcome these technical difficulties, we decided to perform cell treatments followed by IFA. In all these experiments we used the detection of tubulin to monitor the quality of our preparations. Indeed, tubulin is the main component of the subcellular corset, which is a typical cytoskeleton network found immediately under the plasma membrane 24. With an anti-tubulin antibody, the signal looks like a membrane staining in IFA (Figure 3B, panel 1, right). The first set of treatment consisted in removing the plasma membrane with the help of a detergent. This method is currently used to analyze parasite cytoskeletons 25,26. As can be seen from Figure 3B (panels 1 and 2), the effect of the detergent (NP40) can be observed on phase contrast images, which constitute a good quality control of the experiment. In these cells, the typical membrane-like staining disappeared completely by using anti-TcoRep, whereas tubulin could still be detected properly (Figure 3B, panel 2, left and right). This indicates that the signal observed previously (Figure 3A, panel 1) could actually correspond to the plasma membrane.

The purpose of the second set of experiment was to remove the external part of membrane proteins. We shaved the parasites by means of a protease treatment. We used trypsin to detach the parasites from the culture flask and presumed that this treatment removed surface proteins 27. In detached trypsin-treated parasites, the TcoClec signal disappears while that of tubulin is still present (Figure 3B, panel 3). Phase contrast images show that the shape of the cells is not altered by the treatment. After incubation with trypsin, parasites were still alive and could be put back into culture. Interestingly, the TcoClec signal was absent 5 h later and reappeared 14 h later. Altogether these results suggest not only that TcoClecs are plasma membrane proteins but also that they are exposed at the cell surface. Finally, as glycosylation sites could be predicted, cell lysates from *T. congolense* BSF were PNGase-treated and analyzed to see if these proteins are truly post-translationally modified. Figure 3C shows that bands shift after glycosidase treatment, indicating that TcoClecs are glycosylated. Quality control of anti-TcoRep was done by using dot blot analysis. On the left panels of Figure 3D, we can see that our antibodies recognize specifically the peptide motif of *T. congolense* but not that of *T. b. brucei*. The right panels depict the same experiment where the anti-TcoRep was pre-incubated with TcoRep peptide (peptide competition assay). As can be seen in the figure, the signal previously observed disappears, which demonstrates the specificity of our antibody.

## DISCUSSION 

In our study, we identified a new family of membrane glycoproteins containing a putative CLTD with members expressed at the surface of *T. congolense* BSF. This is of particular importance because, to our knowledge, only a few surface molecules have been described so far.

Interestingly, this gene family already appeared in the cell-surface phylome published by Jackson *et al.* 14 and was denominated « lectin-like membrane protein » (Fam77). Recently, another study focused on this gene family in *T. b. brucei *15. The authors provided a very complete and detailed phylogenetic analysis confirming the homogeneity in African trypanosomes. Interestingly, they are localized in the ER in *T. b. brucei*, whereas they are membrane surface-exposed in *T. congolense*. This could reflect species peculiarities such as tissue distribution or pathogenicity.

In non metazoan organisms, CTLD-containing proteins are involved in adhesion to the host 28. In African trypanosomes, the molecules implicated in host contact are not well-documented. Moreover, as pathogenicity and parasite behavior are different from one species to another there is no strictly conserved mechanisms that could solve the problematic in all African trypanosomes. This striking fact is exemplified by the differences between *T. congolense*, which adheres to endothelial cells and red blood cells, and *T. b. brucei*, which does not. Here, proteins involved in host cell adhesion are unknown. It has been hypothesized that candidates should bind to sugars 9, but this has to be experimentally proven. Concerning *T. b. brucei*, it is even more complicated. These parasites circulate freely in blood vessels but can also invade tissues 29,30,31,32. At one point they should first adhere then penetrate 33. There is a lack of literature concerning the molecules involved in these processes. Moreover, the expression and localization of the proteins involved in these events should be highly controlled. This can also be the case in the insect vector, where the journey from the gut to the salivary glands is long and necessitates at some point adhesion to the fly tissues. We are currently investigating whether these proteins could be implicated in adhesion, and the *T. congolense* model is well-suited to address that question.

*T. congolense *is the main causative agent of animal trypanosomiasis, arguably the most important livestock disease in Africa, due to its devastating effects on livestock production across sub-Saharan Africa. Important limitations exist concerning diagnosis and treatments 34. Glycoproteins are antigenic and are used in a number of diagnostic tests. For example, the serodiagnosis of sleeping sickness detects antibodies against a variant surface glycoprotein 35 but does not work with animal trypanosomes. As the TcoClecs described in this paper should face the host’s immune system, they could be used as a diagnostic or therapeutic target.

## MATERIALS AND METHODS

### *In silico* identification of TcoClec genes in *T. congolense*

First, we mined the *T. b. brucei* genome with the Tritryp website (Tritrypdb.org) using « similarity/pattern » and « InterPro domain » corresponding to C-type lectin fold domain. Second, we searched for TMD and signal peptide. Strikingly, all these genes are related, showing primary sequence similarities, and orthologs are found only in African trypanosomes including *T. congolense*. These genes belong to the Fam77 described elsewhere 14,15.

### Cell lines and cell culture

*T. congolense* IL3000 BSF (kindly provided by the International Livestock Research Institute, Nairobi, Kenya) were cultured in Eagle’s Minimum Essential Medium (Sigma) supplemented with 25 mM sodium bicarbonate, 25 mM Hepes, 5.5 mM glucose, 1 mM pyruvate, 0.04 mM adenosine, 0.1 mM hypoxanthine, 0.02 mM thymidine, 0.02 mM bathocuprone, 200 mM glutamine and 20% goat serum at 34°C in a humidified atmosphere containing 5% CO_2_ 36.

U-2 OS cells (human bone osteosarcoma epithelial cells, ATCC® Number: HTB-96, 23) were grown in D-MEM Glutamax (Gibco) supplemented with 10% fetal calf serum and 1% penicillin-streptomycin at 37°C plus 5% CO_2_.

### Plasmid construction and transient transfection of U-2 OS cells

A *TcoClec* ORF (TcIL3000.0.50510) was amplified from *T. congolense* IL 3000 genomic DNA using the two specific primers TcoClec-Fw (*5′-CTCGAGATGAGCAAAGGGAAACACG-3′*) and TcoClecSTOP-rev (*5′-CTACTGAGCAACCGCCGGCGAC-3′*), cloned into pcDNA3.1/CT-TOPO (Invitrogen) and sequenced. Exponentially growing cells were transfected with 250 ng DNA (TcoClec or control) using Lipofectamine 2000 in OPTIMEM (Invitrogen) according to the manufacturer's instructions and processed for IFA 24 h post-transfection. To control transfection efficiency, the pcDNA3.1 Bilbo plasmid was used 22. A transfection without DNA was used as negative control.

### Production of anti-TcoRep antibodies

A peptide corresponding to a sequence found in the repeats (H_2_N-CEELNGTDANETASEELNGTDANETAS-CONH_2_; TcoRep peptide) was synthesized and conjugated to ovalbumin. Antiserum was raised in mice by 4 injections at 15-day intervals of 25 µg of peptides emulsified with complete (first injection) or incomplete Freund’s adjuvant (following boosts).

### Removal of surface proteins by trypsin treatment (« shaving experiment »)

TcIL3000 BSF in suspension were eliminated and adherent parasites were briefly washed twice in 4.5 mL PBS supplemented with glucose (1.8 g/L, gPBS). Cells were then incubated for 3 min with 0.5 mg/mL trypsin solution (SigmaT0134) at RT. To stop the reaction, detached parasites were put at 4°C, collected and washed in gPBS. Culture medium was added; one part was directly analyzed by IFA, and the other was put back in the incubator. Subsequent IFA were performed with these parasites.

### Fluorescence microscopy

#### *T. congolense *BSF

Plastic-adherent trypanosomes grown in culture were scrapped, collected by centrifugation (500 *g*, 5 min, RT) and washed in gPBS.

For whole-cell and « shaving » experiments, trypanosomes were fixed with paraformaldehyde (PFA, 3%) as described elsewhere 22.

For cytoskeleton-extracted cell experiments, parasites were extracted with Pipes buffer (100 mM Pipes pH 6.9, 1 mM MgCl_2_, 0.05% NP40) for 3 min, washed and fixed with PFA (3%).

Slides were incubated with mouse anti-TcoRep (diluted 1:100) or mouse anti-beta tubulin (diluted 1:200) followed by Alexa Fluor 596-conjugated goat anti-mouse secondary antibody (diluted 1:100) (Invitrogen). The nucleus and the kinetoplast were stained with DAPI (10 µg/mL). Cells were viewed using a Zeiss Axio Imager Z1 fluorescent microscope; images were captured using the MetaMorph® software (Molecular Devices) and processed using ImageJ software.

#### U-2 OS cells

Cells grown on coverslips were washed briefly with PBS and fixed in 3% PFA for 20 min. Cells were neutralized in glycine (0.1 M in PBS) and treated for immunolabelling as described 37. Primary antibodies mouse anti-TcoRep (1:250) and anti-calnexin (1:400) were incubated for 1 h in a moist chamber. After three washes, Alexa Fluor 488-conjugated goat anti-mouse secondary antibody and Alexa Fluor 594-conjugated goat anti-rabbit secondary antibody (diluted 1:100) (Invitrogen) were added for 1 h. The nuclei were stained with DAPI (10 µg/mL) and cells were observed as described above.

### N-glycosylation analysis

Parasites were treated with PNGase F (New England Biolabs). 1 x 10^8^ cells were washed in PBS, resuspended in 2% SDS and then heated to 100°C for 10 min. Samples were treated with PNGase F for 3 h at 37°C according to the manufacturer's instructions.

### Western blot and dot blot analysis

For Western blot analysis, total protein lysates of *T. congolense* BSF were separated by SDS-PAGE (4-20% Mini PROTEAN TGX stain-free precast gradient gels, Bio-Rad) and blotted on PVDF filters (Bio-Rad). The membranes were blocked with PBS 5% milk powder for 1 h at RT. For dot blot analysis, 200 ng of peptides were spotted directly on the membranes (nitrocellulose 0.45 µm, Bio-Rad). Primary and secondary antibodies were diluted in TBS (Tris-buffered saline: 10 mM Tris, 150 mM NaCl, pH 7.4) with 0.05% Tween 20 and 5% BSA powder: mouse anti-TcoRep 1:250; rabbit anti-PFR (paraflagellar rod) 1:5000; anti-mouse conjugated to horseradish peroxidase (KPL) 1:5000; or anti-rabbit conjugated to horseradish peroxidase (KPL) 1:10000. Revelations were done using Clarity Western ECL Substrate (Bio-Rad) according to the manufacturer’s instructions.

### Peptide competition assay

Anti-TcoRep was diluted (1:250 in TBS supplemented with Tween 20 and BSA) and equally divided into two tubes; 25 µg of TcoRep peptide was added to one tube, and the equivalent of water was added to the other tube. After 1 h of incubation at RT, the dot blot was pursued as previously described.

### Ethics statement

All animal procedures were carried out in strict accordance with the French legislation (Rural Code articles L 214-1 to L 214-122 and associated penal consequences) and European Union (2010/63/EU) guidelines for the care of laboratory animals and were approved by the Ethical Committee (C2EA-50) of the Centre National de la Recherche Scientifique and by the University of Bordeaux animal care and use committee. All efforts were made to minimize animal suffering.
